# Feasibility of focused cardiac ultrasound during cardiac arrest in the emergency department

**DOI:** 10.1186/s12947-021-00252-3

**Published:** 2021-05-26

**Authors:** Jessica R. Balderston, Alan X. You, David P. Evans, Lindsay A. Taylor, Zachary M. Gertz

**Affiliations:** 1 Department of Emergency Medicine, Virginia Commonwealth University, 1200 E Marshall Street, Richmond, VA 23219 USA; 2 Department of Emergency Medicine, University of California San Diego, 200 W. Arbor Drive, CA 92103 San Diego, USA; 3 Division of Cardiology, Virginia Commonwealth University, 1200 E Marshall Street, VA 23219 Richmond, USA

**Keywords:** Cardiac arrest, Echocardiography, Ultrasound

## Abstract

**Background:**

Focused cardiac ultrasound (FOCUS) can aid in evaluation and management of patients with cardiac arrest, but image quality in this population has been questioned. Our goal was to determine how often adequate imaging can be obtained in cardiac arrest patients.

**Methods:**

We conducted a prospective cohort study to examine the utility of FOCUS in cardiac arrest. All patients who presented to the Emergency Department (ED) in cardiac arrest or who had cardiac arrest while in the ED over 6 months were prospectively identified. FOCUS images were obtained as part of routine clinical care. Patients with images obtained were paired with age- and gender-matched controls who underwent FOCUS for another indication during the study period. Image quality was scored by two blinded reviewers using a 0–4 scale, with a score of ≥ 2 considered adequate.

**Results:**

There were 137 consecutive cardiac arrests, 121 out-of-hospital and 16 in-hospital, during the study period. FOCUS images were recorded in 126 (92%), who were included in the analysis. The average age was 58 years, and 45% were female. Ninety-seven studies (77%) were obtained during advanced cardiac life support while 29 (23%) were obtained after return of spontaneous circulation. The controls were appropriately matched. Of the cardiac arrest studies, 106 (84%) were rated adequate, compared to 116 (92%) in controls (*p* = 0.08). When compared to control FOCUS studies, the scores given to studies of cardiac arrest patients were lower (*p* = 0.001).

**Conclusions:**

FOCUS can reliably be used during cardiac arrest to obtain images adequate to answer clinical questions and guide therapies.

## Background

Resuscitation of patients in cardiac arrest in the Emergency Department (ED) is a common but challenging task with overall poor rates of success [1]. As such, significant effort has been placed on identifying tools or techniques to guide treatment during resuscitation. Focused cardiac ultrasound (FOCUS) may elucidate the etiology of an arrest, enable targeted therapy, and aid in prognosis. Several studies have shown that FOCUS may predict survival by identifying cardiac standstill [2, 3]. Yet the ability to utilize this tool in routine practice is not well known, and some have suggested that poor image quality makes transesophageal echocardiography (TEE) a better modality [4]. Evidence regarding the quality of FOCUS imaging in cardiac arrest is limited and outdated, and nearly all studies have used convenience samples, rather than a prospective, consecutively enrolled patient population [5, 6]. The goal of this study was to determine how often adequate FOCUS imaging can be obtained in cardiac arrest patients.

## Methods

We conducted a prospective cohort study of cardiac arrest patients at an urban academic medical center between August 15^th^, 2019 and February 15^th^, 2020. The study was approved by our institutional review board. The number of cases presenting to our institution during the study period determined the sample size.

Atraumatic cardiac arrest patients were identified prospectively by study personnel who monitored patients entering the ED as part of a quality improvement initiative to improve ultrasound documentation. Patients who arrived in the ED in cardiac arrest or shortly after return of spontaneous circulation (ROSC), or who had cardiac arrest while in the ED, were screened for inclusion. Cardiac arrest was defined as any patient found to be pulseless who received at least one round of cardiopulmonary resuscitation by Emergency Medical Services or in the ED. To ensure no cardiac arrest patients were missed, the electronic medical record was queried for an International Classification of Diseases 10 code of I46.2, I46.8, or I46.9 for all patients presenting to the ED during the study period.

FOCUS images were obtained by resident or attending physicians as part of routine clinical care. All images were obtained using the Sonosite X Porte ultrasound machine (Fujifilm Sonosite, Bethell, WA) and acquired with the 5-1 MHz phased array probe. Before the quality improvement initiative began, residents were trained on techniques to use while obtaining images during cardiac arrest to help decrease pause time and avoid ultrasound when it is not appropriate, such as when a patient is in ventricular tachycardia. Residents were instructed to attempt the subxiphoid view first and record images during pauses in compressions.

We wanted to compare image quality between cardiac arrest patients and stable patients undergoing FOCUS. After the study period was complete, cardiac arrest patients with images obtained were paired with age- and gender-matched controls who underwent elective FOCUS for another clinical indication during the study period. Image quality was evaluated using the following scoring system adapted from Kimura et al.: 0 = no image obtained, 1 = only cardiac motion detected, 2 = chambers and valves grossly resolved with the left ventricle and posterior epicardium visible, 3 = endocardium and wall thickness seen but incomplete, and 4 = greater than 90% of endocardium and valve motion seen, with a score of ≥ 2 considered adequate [7]. All studies were scored by two blinded ultrasound fellowship-trained emergency physician reviewers. When a disagreement between scorers was present, a score or interpretation by a third reviewer was obtained. Studies were interpreted for left ventricular function and presence of a pericardial effusion. Left ventricular function was visually assessed as either normal (ejection fraction ≥ 55%), reduced (ejection fraction 30–54%), severely reduced (ejection fraction < 30%), or cardiac standstill [8]. Pericardial effusions were graded as small, moderate, or large; trace effusions (those only visualized during systole) were considered negative. The studies were assessed for the number of views obtained, including subxiphoid, parasternal long axis, parasternal short axis, and apical four chamber views.

Means, proportions, and associated 95% confidence intervals (CIs) were calculated as appropriate. Continuous variables were compared using a t-test or Mann–Whitney test as appropriate, while categorical variables were compared using a Chi-Square test. A linear weighted kappa coefficient was used to measure inter-rater reliability in cardiac arrest patients. All statistical analyses were performed with SPSS ver. 24 (SPSS, Chicago, IL, USA). All significance tests were 2-sided, and *p* < 0.05 was considered significant.

## Results

There were 137 consecutive cardiac arrest patients, 121 out-of-hospital and 16 in-hospital, during the study period. FOCUS images were recorded in 126 (92%), and these patients were included in the analysis (Table 1). Control patients were appropriately matched. In the 11 patients with no images recorded, 4 had no ultrasound attempted while in 7 there was documentation of an ultrasound attempt. In 6 of these 7 patients, the ultrasound was noted to be adequate for interpretation. The presenting rhythm of arrest patients included in the analysis was ventricular fibrillation or tachycardia in 19 (15%) patients, pulseless electrical activity in 49 (39%) patients, asystole in 49 (39%) patients, and unknown in 9 (7%) patients. In cardiac arrest FOCUS studies, 10 (8%) were noted to have a pericardial effusion, including one large pericardial effusion, 2 moderate effusions, and 7 small effusions. Left ventricular function was noted to be normal in 25 (20%), reduced in 14 (11%), severely reduced in 29 (23%), and cardiac standstill in 58 (46%). During the resuscitation, either in hospital or before arrival, 32 (25%) patients underwent defibrillation and 120 (95%) received epinephrine. Fifty-five (44%) survived to leave the ED, and 16 (13%) survived to hospital discharge.

**Table 1:** Patient characteristics

	Total Patients (*n* = 126)	Adequate Images (*n* = 106)	No Adequate Images (*n* = 20)	*P* value
Age, years	60 (47–72)	62 (47–72)	57 (49–66)	0.52
Gender (Female)	58 (46%)	51 (48%)	7 (35%)	0.28
Out of Hospital Arrest	111 (88%)	91 (86%)	20 (100%)	0.07
FOCUS Obtained during ACLS	97 (77%)	79 (75%)	18 (90%)	0.13
Total Duration of CPR (minutes)	32 (10–46)	30 (10 -45)	42 (29 -50)	0.16
CPR Performed in the ED	107 (85%)	90 (85%)	19 (95%)	0.31

*P* values compare those with adequate images to those with no adequate images. Data are presented as median (interquartile range) and number (percent) where applicable

*FOCUS* Focused cardiac ultrasound, *ACLS* Advanced cardiac life support, *CPR* Cardiopulmonary resuscitation, *ED* Emergency department

**Fig. 1:**
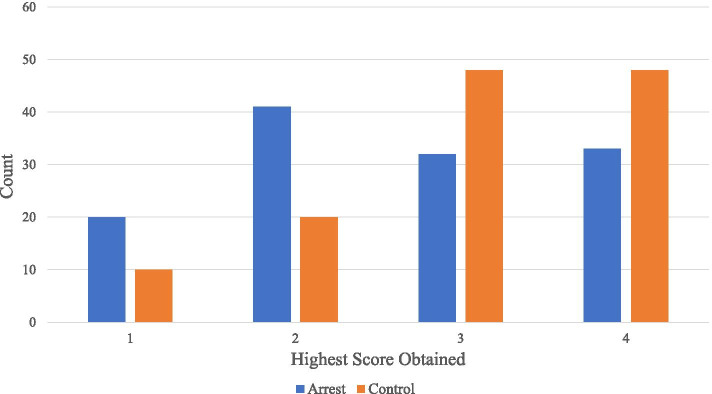
Highest Scores Obtained by Cardiac Arrest and Control Studies

Of the 126 cardiac arrest patients with recorded images, each study had on average 1.6 views recorded and 106 (84%) had at least one view rated adequate. The control studies had on average more views recorded (3.2, *p* < 0.001 compared to arrest patients), but a similar percentage with at least one view rated adequate (92%, *p* = 0.08). Using the 4-point scale, FOCUS studies of control patients received higher quality ratings than those of cardiac arrest patients (Fig. 1, *p* = 0.001). Inter-rater reliability between the two study reviewers was substantial (linear weighted Kappa: 0.66; 95% CI 0.60–0.72). The FOCUS view with the highest average score in cardiac arrest studies was the subxiphoid view.

Out of all 126 arrest patients with imaging, the two reviewers agreed on whether the patient had cardiac activity in 96% of cases. There were 5 cases where one reviewer interpreted the study as cardiac standstill and the other interpreted it as reduced or severely reduced. Of these 5 studies, 4 were scored as adequate and one was scored as inadequate. The reviewers agreed on the presence of pericardial effusion in 92% of patients. This was similar to the level of agreement in control patients (97%, *p* = 0.17). The reviewers agreed on all three patients that were noted to have moderate or large pericardial effusions. At least one of the reviewers could not make an adequate assessment for pericardial effusion in 8 patients with cardiac arrest and 2 control patients (*p* = 0.10).

## Discussion

The goal of this study was to determine how often adequate FOCUS imaging can be obtained in cardiac arrest patients. Among patients presenting to an urban academic medical center over a 6-month period, images were recorded in 92% of cardiac arrest patients, and 84% of those had at least one image rated adequate. This was not significantly different than the number of control patients with at least one adequate image. This study is the first to demonstrate that it is both possible to regularly obtain images during cardiac arrest management and that the adequacy of these images does not differ from those used in non-emergent settings.

Prior studies measuring the efficacy of FOCUS have been performed but have failed to assess a comparable population or used technology that is now considered outdated. Heidenreich et al. were among the first to study this topic and reported that FOCUS provided adequate imaging in only 36% of cases [5]. However, this publication was released in 1995 and ultrasound technology has improved since then. Additionally, their study population consisted primarily of patients experiencing unexplained hypotension rather than cardiac arrest. Using data from the Real-time Evaluation and Assessment Sonography Outcomes Network registry, Gaspari et al. reported that FOCUS was effective in obtaining images that could differentiate between organized and disorganized electrical activity in 76% of patient with a pulseless electrical activity arrest [6]. However, this registry did not include all patients who presented in cardiac arrest during their study period, only those who underwent FOCUS as a part of their resuscitation. Such a convenience sample cannot inform us of how often adequate images can be obtained when FOCUS is deployed routinely. In our study, images were recorded in over 90% of patients, with adequate images obtained in the vast majority of patients. This suggests that FOCUS can be employed routinely by providers treating patients with cardiac arrest.

Ours is the first study to attempt to use a definition for adequate FOCUS imaging during cardiac arrest. We used a definition for adequate based on that developed by Kimura et al. [7]. While this semi-objective scoring system is an improvement compared to an operator’s purely subjective assessment of whether FOCUS images are sufficient, further study is required to link this definition adequate imaging to clinical outcomes and ensure its utility. It is a positive sign that there was a very strong correlation between image interpreters as to the presence or absence of cardiac activity in our study, suggesting the vast majority of studies were likely adequate to answer the study question. Subsequent studies that evaluate for a difference between image quality in FOCUS compared to TEE would do well to incorporate some scoring method, and we feel that the definition of adequacy used in our study would be reasonable.

Multiple studies have shown that FOCUS during cardiac arrest may lead to longer pause times during cardiopulmonary resuscitation compared to when FOCUS is not used [2, 3]. Yet this may be alleviated with role clarity. Pauses are longer when the person leading the resuscitation is also performing the ultrasound [2]. In a prospective study by Lien et al., it was shown that pause duration was almost always < 10 s, the recommended maximum length of a pulse check, when the person performing the ultrasound was an independent member of the team [9]. Pause duration is also shorter when the provider obtaining the FOCUS exam has a higher level of ultrasound training [2]. This suggests that with best practices FOCUS may be used during cardiac arrest without unacceptable pauses.

Some have argued for the use of TEE rather than transthoracic FOCUS in cardiac arrest because TEE images are higher quality [4]. Inherent in this argument is the assumption that regular use of TEE will be as successful as FOCUS in obtaining images. Our study shows that providers can obtain adequate cardiac images in the majority of cardiac arrest cases using FOCUS, while to date there have been no studies that address the ability of the emergency physician to routinely obtain TEE images in consecutive patients. Roadblocks in TEE imaging include the patient having a secure airway, the ability to pass the probe, and the ability to obtain images once the probe is in the correct location. Until a prospective study of TEE on consecutive patients in the ED is performed, recommendations regarding its use should be limited. Ultimately, a randomized trial comparing the two modalities may be necessary.

While TEE images are likely higher quality than FOCUS images, it is uncertain whether higher quality images are required to meet the clinical need in cardiac arrest patients. The main diagnostic goals of echocardiography during cardiac arrest are to identify cardiac activity and to help determine the etiology of the arrest [10]. With limited FOCUS in our study there was agreement on the presence or absence of cardiac activity by both reviewers in 96% of cases, with the only disagreements arising when one reviewer interpreted the study as cardiac standstill and the other as reduced or severely reduced left ventricular function. Thus, a determination on the presence or absence of cardiac standstill may be possible in nearly all patients studied. Although the cardiac arrest patients in our study had significantly fewer views obtained when compared to controls, we suspect that the providers obtained the images needed to make a clinical decision regarding cardiac standstill and concluded the study to move on to other aspects of the resuscitation effort.

Our study has several limitations. It represents a single-center experience. Despite our quality improvement initiative, we were not able to record images in 100% of the cardiac arrests that presented during the study period. Still, images were obtained more frequently than in any previous study, and we believe a sufficient number of patients were included to answer the clinical question [9].

## Conclusions

Transthoracic FOCUS can reliably be used in cardiac arrest patients to obtain images adequate to answer clinical questions and guide resuscitative efforts.

## Data Availability

The datasets generated and/or analyzed during the current study are not publicly available due to institutional restrictions but are available from the corresponding author on reasonable request.

## Abbreviations

ED: Emergency department
FOCUS: Focused cardiac ultrasound
ROSC: Return of spontaneous circulation
TEE: Transesophageal echocardiography
